# Dantrolene improves left ventricular diastolic property in mineralcorticoid-salt-induced hypertensive rats

**DOI:** 10.1016/j.bbrep.2023.101449

**Published:** 2023-03-01

**Authors:** Junya Nawata, Takeshi Yamamoto, Shinji Tanaka, Yasutake Yano, Tomoyuki Uchida, Shohei Fujii, Yoshihide Nakamura, Takeshi Suetomi, Hitoshi Uchinoumi, Tetsuro Oda, Shigeki Kobayashi, Masafumi Yano

**Affiliations:** Department of Medicine and Clinical Science, Division of Cardiology, Yamaguchi University Graduate School of Medicine, 1-1-1 Minamikogushi, Ube, Yamaguchi, 755-8505, Japan

**Keywords:** Dantrolene, Calmodulin, Hypertrophy, HFpEF

## Abstract

Left ventricular (LV) diastolic dysfunction is increasingly common in heart failure with preserved ejection fraction (HFpEF), and new drug therapy is desired. We recently reported that dantrolene (DAN) attenuates pressure-overload induced hypertrophic signaling through stabilization of tetrameric structure of cardiac ryanodine receptor (RyR2). Because cardiac hypertrophy substantially affects LV diastolic properties, we investigated the effect of DAN on LV diastolic properties in mineralocorticoid-salt–induced hypertensive rat model exhibiting the HFpEF phenotype.

Male Sprague-Dawley (SD) rats (8 weeks old) received an uninephrectomy (UNX), subcutaneous implantation of a 200 mg pellet of deoxycorticosterone acetate (DOCA), and 0.9% NaCl water (UNX + DOCA-salt). UNX, a control pellet, and water without NaCl served as controls (UNX control). The effect of oral administration of 100 mg/kg/d DAN was examined in UNX control and UNX + DOCA-salt groups (UNX + DAN and UNX + DOCA-salt + DAN).

UNX + DOCA-salt treatment resulted in mild hypertension. Chronic administration of DAN to UNX + DOCA-salt rats (UNX + DOCA-salt + DAN) did not affect blood pressure. DAN treatment increased the mitral annular early relaxation velocity in the UNX + DOCA-salt group. The size of cardiomyocytes increased in the UNX + DOCA-salt group, whereas the increase was suppressed by DAN treatment. LV fibrotic area was significantly smaller in the UNX + DOCA-salt + DAN group than in the UNX + DOCA-salt group (2.0 ± 0.2% vs 4.0 ± 0.4%). The LV chamber stiffness significantly increased in the UNX + DOCA-salt group, whereas the increase was suppressed by DAN treatment. DAN treatment normalized the CaM-RyR2 interaction and inhibited aberrant Ca^2+^ release.

DAN improved left ventricular diastolic properties with respect to both myocardial relaxation and chamber stiffness. DAN may be a new treatment option for HFpEF.

## Highlights

• Dantrolene improved relaxation and LV stiffness in hypertensive rats.
• Dantrolene improved aberrant Ca2 release in hypertensive rats.
• Dantrolene increased RyR2 bound CaM in hypertensive rats.
• Dantrolene may be a new treatment option for HFpEF.

## 1 Introduction

Heart failure with preserved ejection fraction (HFpEF) is becoming increasingly prevalent and responsible for a significant increase in the number of heart failure patients [1,2]. Empagliflozin has been recently shown to improve the rates of cardiovascular death and hospitalization attributed to HFpEF [3]. However, the optimal therapy targeting diastolic dysfunction in HFpEF, which is often caused by cardiac hypertrophy, has not yet been established [4].

As an important mechanism for triggering pressure-overload induced hypertrophic signaling, we previously showed that the dissociation of calmodulin (CaM) from ryanodine receptor (RyR2) and its subsequent translocation to the nucleus is critical for the development of pressure-overload hypertrophy [5]. Then, we further reported that the dissociated CaM from RyR2, along with the aberrant Ca^2+^ release, activated two Ca^2+^-dependent hypertrophic signaling pathways, namely the CaM kinase II (CaMKII)–histone deacetylase (HDAC) and calcineurin–nuclear factor of activated T cells (NFAT) pathways [6]. Taken together, it is strongly suggested that the defective interaction of CaM with RyR2 is a key factor in driving cardiac hypertrophy via the two major hypertrophic signaling pathways. In this regard, we further showed that dantrolene (DAN) prevents the dissociation of CaM from RyR2 in heart failure [7], pulmonary arterial hypertension [8], and catecholaminergic polymorphic ventricular tachycardia [9,10].

Based on these concepts, we examined whether DAN could improve diastolic dysfunction caused by cardiac hypertrophy and prevent the development of heart failure in mineralocorticoid-salt–induced hypertensive rat model exhibiting the HFpEF phenotype.

## 2 Methods

### 2.1 Chemicals and antibodies

DAN and deoxycorticosterone acetate (DOCA) were purchased from Fuji Film Wako Chemicals, Tokyo. The antibodies used included anti-CaM (EP799Y, abCaM) and anti-RyR (C3-33, Sigma-Aldrich). DOCA pellets were prepared according to a previous report [11], with minor modifications. Briefly, 2.0 mL of Sylgard 184 Silicone part A was placed in a weigh boat along with 200 mg of DOCA and mixed thoroughly with a spatula. Then, 0.2 mL of curing agent was added to the silicone mixture and mixed thoroughly, and the mixture was covered with pre-labeled aluminum foil. The DOCA pellets were cured at room temperature for 24 h and then refrigerated at 4 °C until the day of the surgical implantation procedure.

### 2.2 Animal model

This study conformed to the ARRIVE guidelines and the Guide for the Care and Use of Laboratory Animals published by the US National Institute of Health (NIH Publication No. 85–23, revised 1996). All experimental protocols were approved by the Animal Ethics Committee of the Yamaguchi University School of Medicine. Animal care and protocols were in accordance with the guidelines of the Animal Ethics Committee of the Yamaguchi University School of Medicine.

**Fig. 1:**
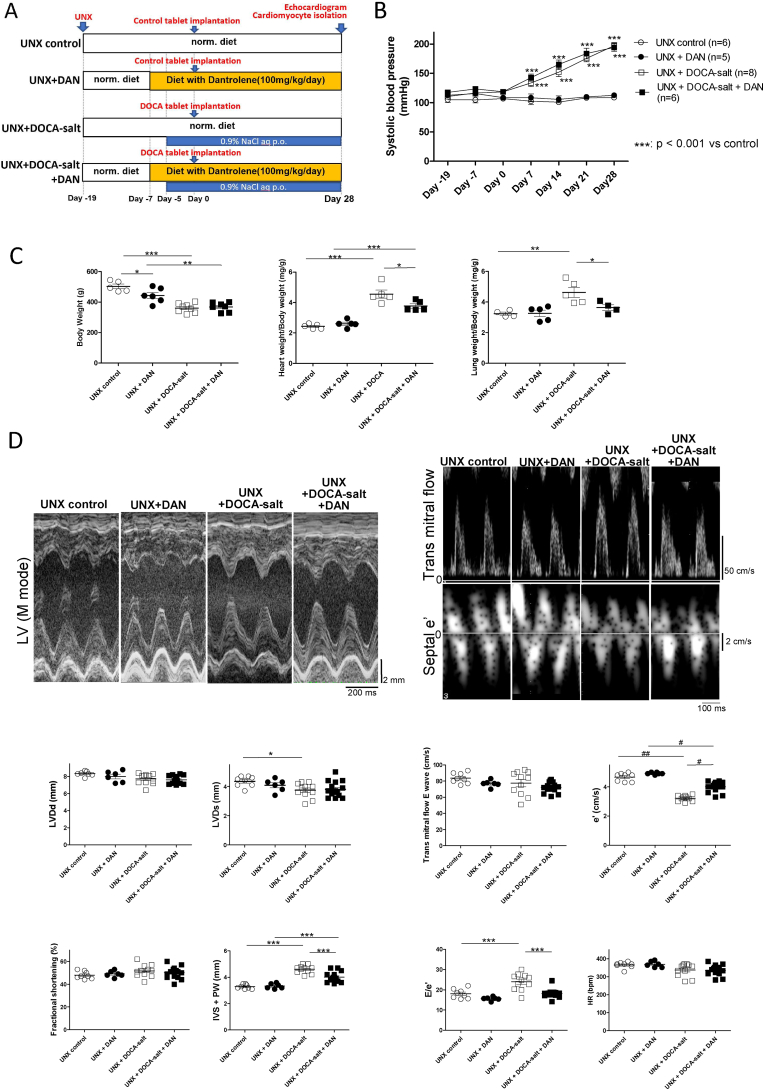
Research design, blood pressure, cardiac and lung weights, echocardiograms. (A) Study design of DOCA-salt induced hypertensive rat. (B) Blood pressure of the rats in each group (n = 5–8). (C) Body weight, heart weight and lung weight in each group (n = 4–8). (D) Representative echocardiograms, and the summarized data (n = 6–15). ***P < 0.001 vs UNX control (two-way ANOVA with a post-hoc Bonferroni test) for B. *P < 0.05, **P < 0.01, ***P < 0.001 (one-way ANOVA with a post-hoc Tukey's test). #P < 0.05, ##P < 0.01, ###P < 0.001 (KW with a post-hoc Dunn's test). LV, left ventricular; LVDd, left ventricular diastolic dimension; LVDs, left ventricular systolic dimension; IVS, intra-ventricular septum; PW, posterior wall.

All experiments were performed using male 8-week-old Sprague-Dawley rats (200–240 g). As shown in Fig. 1A, the UNX + DOCA-salt group received an uninephrectomy (UNX) on day −19. Drinking water was exchanged with 0.9% sodium chloride in water on day −7. The rats were implanted with 200 mg DOCA tablets on day 0. The UNX control group was implanted with control tablets, and regular drinking water was provided until day 28. In the UNX + DAN and UNX + DOCA-salt + DAN groups, DAN (Fuji Film Wako Chemicals, Tokyo, Japan) was orally administered at a dosage of 100 mg/kg/d using a feeding apparatus (Roden CAFÉ; Oriental Yeast Co., Ltd., Tokyo, Japan) starting on day −7. None of the animals exhibited any signs of DAN toxicity.

### 2.3 Blood pressure measurement

The blood pressure of the rats was measured weekly by the tail-cuff method, using a non-invasive blood pressure analysis system (Softron BP-98A, Tokyo, japan).

### 2.4 Echocardiography

Cardiac function was analyzed on day 28 using an F37 ultrasound machine (Hitachi Medical, Netherlands) equipped with a 4–14 MHz linear probe (UST-5417) and a 3.8–7.5 MHz sector probe (UST-5298). Using transthoracic echocardiography under light anesthesia, the left ventricular (LV) end-diastolic diameter, LV end-systolic diameter, percent fractional shortening, and thickness of the interventricular septum (IVS) and posterior wall (PW) were measured. Transmitral flow was obtained using pulsed-wave Doppler imaging with a linear probe. The mitral annular early relaxation velocity (e′) was determined using tissue Doppler imaging with a sector probe.

### 2.5 Histological analysis of the heart

On day 28, immediately after death, the hearts were fixed in 10% formalin. Four-chamber sections were selected for morphometric analysis. The extent of fibrosis was analyzed using Picro Sirius red stain. Red-stained areas were measured using ImageJ software.

### 2.6 Measurement of LV chamber stiffness

LV chamber stiffness was measured on day 28 as described previously [12], with some modifications. After cardiac arrest was induced with potassium chloride, the heart was quickly removed, and an incision was made at the right ventricular (RV) free wall to release pressure. A catheter attached to the pressure transducer and infusion pump was passed through the LV. After gently aspirating the LV cavity to remove residual blood, normal saline was infused into the LV at a rate of 0.70 mL/min using a syringe infusion pump (KD Scientific, Holliston, MA) until the pressure rose to 40 mm Hg, while recording the pressure. This procedure was performed twice within 10 min of cardiac arrest.

### 2.7 Isolation of cardiomyocytes

Cardiomyocytes were isolated on day 28 as described previously [8], with some modifications. Briefly, the rats were anesthetized with pentobarbital sodium (100 mg/kg of body weight, i.p.), intubated, and ventilated with ambient air. An incision was made in the chest, and the heart was quickly removed. Retrograde perfusion was initiated using collagenase-free 5% CO_2_/95% O_2_-bubbled minimal essential medium (Sigma, St. Louis, MO) under constant flow via the aorta, followed by perfusion with same buffer supplemented with 50 μmol/L Ca^2+^, 0.5 mg/mL collagenase B, 0.5 mg/mL collagenase D, and 0.02 mg/mL protease type XIV for 120 min. LV myocardium was minced with scissors in a fresh collagenase-containing buffer and the rod-shaped cardiomyocytes were prepared. The Ca^2+^ concentration was then gradually increased to a final concentration of 1 mmol/L by increasing the volume of the incubation medium (from 50 μmol/L to 100 μmol/L, 300 μmol/L, 600 μmol/L, and 1 mmol/L). The isolated cardiomyocytes were transferred to laminin-coated glass culture dishes and incubated for a few hours at 37 °C in a 5% CO_2_/95% O_2_ atmosphere.

### 2.8 Immunofluorescence analysis of endogenous RyR2-bound CaM

Immunofluorescence staining of isolated cardiomyocytes was performed as described previously [8]. Isolated cardiomyocytes were fixed in 4% paraformaldehyde for 5 min. Thereafter, the cardiomyocytes were incubated overnight at 4 °C with anti-CaM (EP799Y, abCaM, 1:250 dilution) and anti-RyR2 antibodies (C3-33, Sigma-Aldrich, 1:500 dilution) in 1% bovine serum albumin and 0.5% Triton X-100. They were then labeled with 1:300 diluted Alexa 488-conjugated goat anti-rabbit and Alexa 633-conjugated goat anti-mouse secondary antibodies. The sarcomere-related periodic increase in Alexa 633 and Alexa 488 fluorescence intensity from the baseline was integrated with respect to the longitudinally selected distance (∼25 μm), and the value was then divided by the distance. The mean value of a single sarcomere-related increase in fluorescence intensity was calculated as the arbitrary amount of RyR2 and RyR2-bound CaM.

### 2.9 Analysis of Ca^2+^ sparks and sarcoplasmic reticulum (SR) Ca^2+^ content

Ca^2+^ sparks in intact cardiomyocytes were measured using a laser-scanning confocal microscope (LSM-510, Carl Zeiss), as described previously [8]. In brief, the intact cardiomyocytes were loaded with Fluo-4 AM (20 μM; Molecular Probes) for 20 min at 24 °C. Line-scan mode was used, in which a single cardiomyocyte was scanned repeatedly along a line parallel to the longitudinal axis, while avoiding the nuclei. To assess Ca^2+^ sparks, cardiomyocytes were stimulated until the Ca^2+^ transient reached a steady state at 1 Hz for 30 s. Stimulation was then stopped, and Ca^2+^ sparks were recorded during the subsequent 10 s of rest. Data were analyzed using SparkMaster, an automated analysis program that enables rapid and reliable spark analysis [13]. The variables analyzed included general image parameters, such as the number of detected sparks and spark frequency. To assess the SR Ca^2+^ content, caffeine (10 mM) was rapidly perfused to discharge SR-loaded Ca^2+^.

### 2.10 Monitoring of Ca^2+^ transients in cardiomyocytes

Ca^2+^ transients in isolated cardiomyocytes were measured as previously described [8]. Isolated ventricular cardiomyocytes were incubated with 20 μM Fluo-4 acetoxymethyl ester for 20 min at 24 °C and washed twice with Tyrode's solution. All experiments were conducted at 24 °C. Intracellular Ca^2+^ measurements in cells stimulated with a field electric stimulator (IonOptix, MA, Westwood) were acquired using a laser-scanning confocal microscope (LSM-510, Carl Zeiss, Germany). The relative occurrence of spontaneous Ca^2+^ release upon cessation of stimulation at 1 Hz, 2 Hz, 3 Hz, 4 Hz, and 5 Hz was measured a fluorescent digital microscope (BZ9000, Keyence, Osaka, Japan).

### 2.11 Statistical analyses

One-way analysis of variance with a post-hoc Tukey's test was used for statistical comparison of the four groups with a normal distribution. The Kruskal-Wallis test with a post-hoc Dunnett's test was used for statistical comparison of the four groups without a normal distribution. Two-way ANOVA with a post-hoc Bonferroni test was used for BP measurements which have treatment and time point two factors. P < 0.05 is considered to be significant.

## 3 Results

### 3.1 DAN increased e' and reduced LV wall thickness without changing blood pressure

As shown in Fig. 1B, Blood pressure in the UNX + DOCA-salt and UNX + DOCA-salt + DAN groups increased similarly after 7 days. However, the LV weight and wall thickness (IVS + PW) significantly decreased in the UNX + DOCA-salt + DAN group compared with the UNX + DOCA-salt group (Fig. 1C and D). As shown in Fig. 1D, e' in the UNX + DOCA-salt group significantly decreased compared with the UNX control group. DAN treatment significantly increased e'. E/e' and lung weight were also improved by DAN treatment (Fig. 1C and D).

### 3.2 DAN reduced LV fibrosis and chamber stiffness in the UNX + DOCA-salt–induced hypertensive rats

**Fig. 2:**
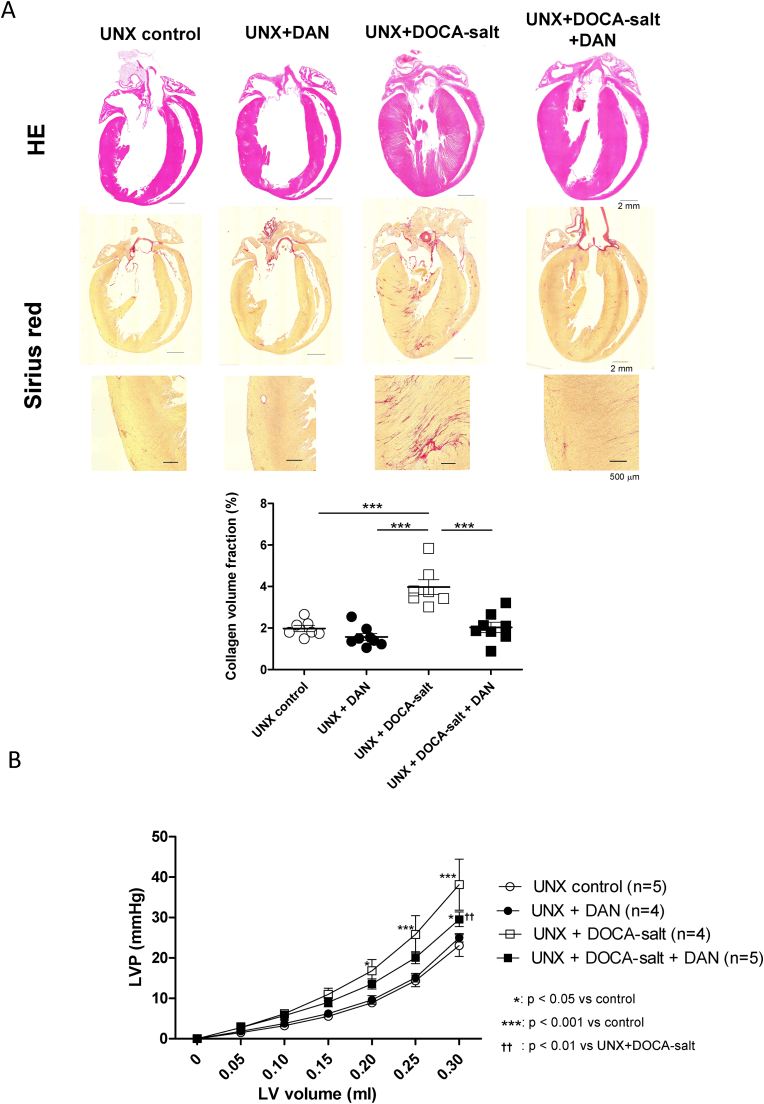
DAN inhibited the development of LV hypertrophy and decreased LV chamber stiffness in UNX + DOCA-salt induced hypertensive rats. (A) Representative images of long-axis sections of HE- or Serius red-stained hearts and the RV tissues. Fibrotic areas of the LV wall obtained from Serius red-stained LV tissue are shown in the lower panel (n = 7–8) (B) Static LV pressure-volume relationship as a parameter of LV chamber stiffness (n = 4–5) *P < 0.05, **P < 0.01, ***P < 0.001 (one-way ANOVA with a post-hoc Tukey's test) for A. *P < 0.05, ***P < 0.001 vs UNX control (two-way ANOVA with a post-hoc Bonferroni test) for B. ††P < 0.05 vs UNX + DOCA-salt (two-way ANOVA with a post-hoc Bonferroni test) for B. (For interpretation of the references to colour in this figure legend, the reader is referred to the Web version of this article.)

The fibrotic area increased in the UNX + DOCA-salt group compared with the UNX control group (Fig. 2A). This increase was inhibited in the UNX + DOCA-salt + DAN group (Fig. 2A). LV chamber stiffness increased in the UNX + DOCA-salt group compared with the UNX control group (Fig. 2B), and DAN treatment prevented this increase.

### 3.3 DAN inhibited hypertrophy and improved relaxation in LV cardiomyocytes prepared from the UNX + DOCA-salt–induced hypertensive rats

**Fig. 3:**
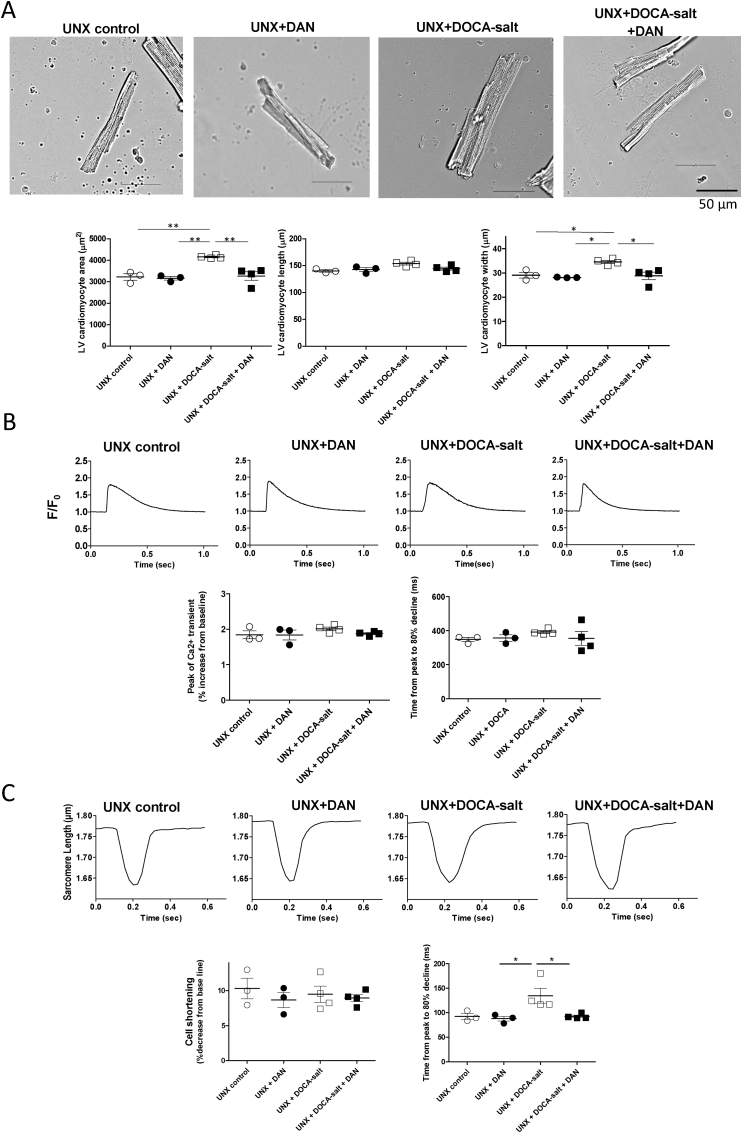
Morphology, Ca^2+^ transient and cell shortening in intact cardiomyocytes. (A) Representative images of isolated cardiomyocytes, and the summarized data of cell width, cell length and cell area (n = 89–159 cells from 3 to 4 hearts). (B) Representative recordings of fluo-4 AM fluorescence signal at a pacing rate of 1 Hz, and the summarized data (n = 4–15 cells from 3 to 4 hearts). (C) Representative recordings of sarcomere length at a pacing rate of 2 Hz, and the summarized data (n = 9–15 cells from 3 to 4 hearts). *P < 0.05, **P < 0.01, ***P < 0.001 (one-way ANOVA with a post-hoc Tukey's test).

Consistent with the *in vivo* data, LV cardiomyocyte hypertrophy, observed in the UNX + DOCA-salt group, was inhibited in the UNX + DOCA-salt + DAN group (Fig. 3A). Neither the peak Ca^2+^ transient nor the time from peak to 80% decline showed significant differences among the four groups (Fig. 3B). On the other hand, the time from peak sarcomere shortening to 80% decline was prolonged in the UNX + DOCA-salt cardiomyocytes, but in the UNX + DOCA-salt + DAN cardiomyocytes, it restored to a baseline level observed in the control groups (Fig. 3C). These results suggest that hypertrophy-induced exacerbation of cardiomyocyte relaxation could be ameliorated by DAN.

### 3.4 DAN suppressed aberrant Ca^2+^ release and spontaneous Ca^2+^ transients (SCaTs) in LV cardiomyocytes prepared from the UNX + DOCA-salt–induced hypertensive rats

**Fig. 4:**
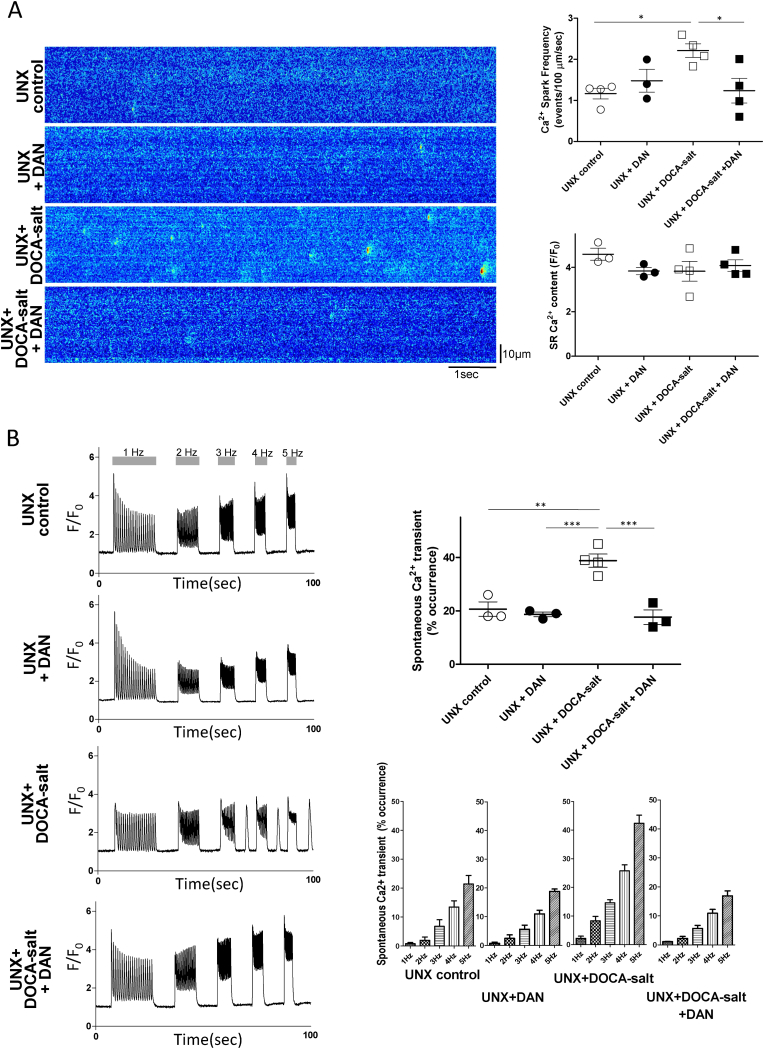
Ca^2+^ spark, sarcoplasmic reticulum (SR) Ca^2+^ content, and spontaneous Ca^2+^ transient (SCaT) in intact cardiomyocytes. (A) Representative recordings of spontaneous Ca^2+^ sparks. On the left, recordings of line-scan image of fluo-4 AM fluorescence in cardiomyocytes; on the right, the summarized data (n = 10–15 cells from 3 to 4 hearts). The SR Ca^2+^ content was measured as follows: caffeine-induced Ca^2+^ transients were measured by first applying a stimulation train at 2 Hz, and then by rapidly switching from the superfusing solution to a solution containing 20 mM caffeine for 5–6 s. The summarized data is indicated at right lower panel (n = 41–114 cells from 3 to 4 hearts). (B) On the left, representative traces of spontaneous Ca^2+^ transients (SCaTs); on the right, the summarized data as the incidence of SCaTs (n = 45–84 cells from isolated cardiomyocytes of 3–4 hearts as indicated). Arrows indicate SCaTs. The SCaTs were induced after cessation of pacing at 1, 2, 3, 4, and 5 Hz of stimulation. Values for individual rats are plotted with mean ± standard error of mean. *P < 0.05, **P < 0.01, ***P < 0.001(ANOVA with a post-hoc Tukey's test) for A.

Ca^2+^ spark frequency increased in the UNX + DOCA-salt cardiomyocytes, but not in the UNX + DOCA-salt + DAN cardiomyocytes (Fig. 4A). SR Ca^2+^ content showed no significant difference between the UNX + DOCA-salt cardiomyocytes and in the UNX + DOCA-salt + DAN cardiomyocytes (Fig. 4A). The frequency of spontaneous Ca^2+^ transient (SCaT) increased in the UNX + DOCA cardiomyocytes but not in the UNX + DOCA-salt + DAN cardiomyocytes (Fig. 4B).

### 3.5 Amount of endogenous CaM bound to RyR2 decreased in cardiomyocytes prepared from the UNX + DOCA-salt–induced hypertensive rats

**Fig. 5:**
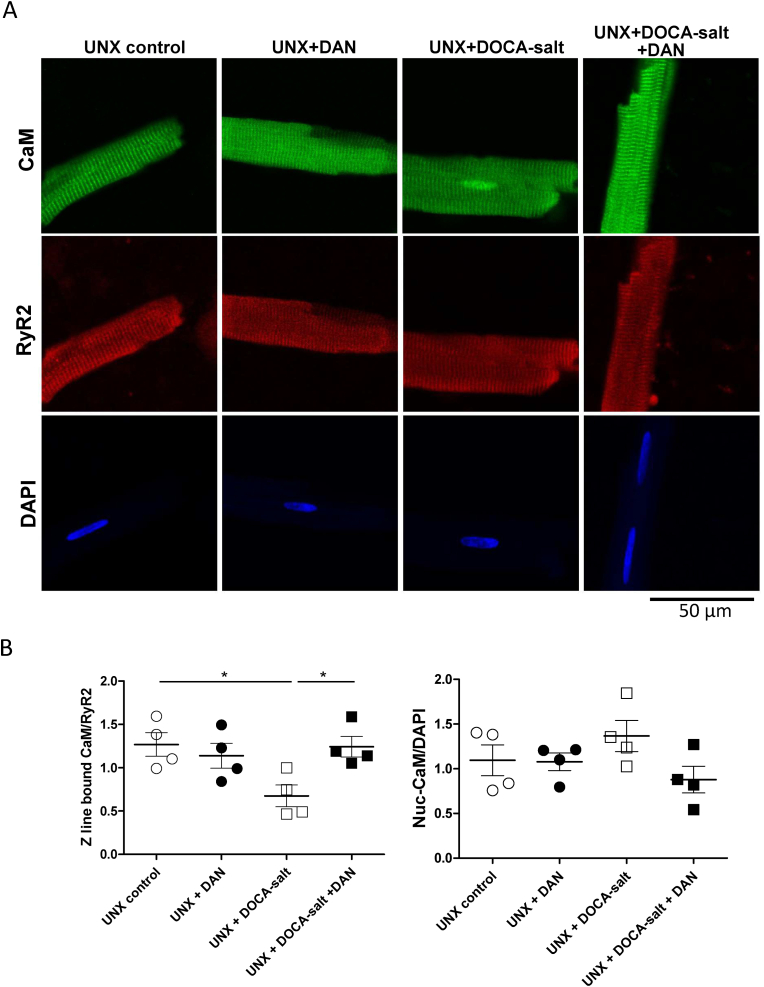
Localization and association with RyR2 of endogenous CaM in cardiomyocytes. (A) Representative images of endogenous CaM, which is co-localized with RyR2 in isolated cardiomyocytes; immunostaining of CaM (shown in green); immunostaining of RyR2 (red). (B) Summarized data of Z line-bound and nuclear CaM. The immunofluorescence signal of Z line-bound CaM was divided by that of RyR2, normalized to control (baseline of Sham), and expressed as ratios. The immunofluorescence signal of nuclear CaM was divided by that of DAPI for nuclear staining, normalized to control (baseline of Sham), and expressed as ratios. Data are presented as mean ± standard error of mean of 3–4 hearts (each plot is the average of 4–5 cells). *P < 0.05, **P < 0.01, ***P < 0.001(ANOVA with a post-hoc Tukey's multiple comparison procedure. (For interpretation of the references to colour in this figure legend, the reader is referred to the Web version of this article.)

The amount of endogenous CaM bound to RyR2 was measured using immunofluorescence analysis. The amount of CaM bound to RyR2 significantly decreased in the UNX + DOCA-salt cardiomyocytes, but not in the UNX + DOCA-salt + DAN cardiomyocytes (Fig. 5AB). The amount of nucleus CaM was increased in UNX + DOCA-salt cardiomyocytes, however the difference was not statistically significant (Fig. 5AB).

## 4 Discussion

The most important finding of this study was that DAN inhibited aberrant Ca^2+^ release by normalizing the CaM-RyR2 interaction, which in turn suppressed LV hypertrophy and fibrosis, thereby ameliorating LV relaxation and chamber stiffness in DOCA salt-induced hypertensive rats.

### 4.1 DAN inhibited the aberrant Ca^2+^ release and improved cardiomyocyte relaxation

Aberrant Ca^2+^ release, identified as Ca^2+^ sparks from RyR2, occurred in our hypertensive rat model. Treatment with 100 mg/kg/d DAN inhibited the Ca^2+^ sparks, which may contribute to amelioration of cardiomyocyte relaxation, as indicated by a decrease in the time from peak sarcomere shortening to 80% decline. Inhibition of the aberrant Ca^2+^ release from the RyR2 could also prevent Ca^2+^-activated hypertrophic signals (see section 4.3) and arrhythmias.

### 4.2 DAN suppressed LV fibrosis associated with decreased passive LV chamber stiffness

The fibrotic area decreased by chronic administration of DAN. In pressure overload-induced LV fibrosis, growth factors such as transforming growth factor β and platelet-derived growth factor are thought to be key factors mediating cardiac fibrosis [14]. A recent report revealed that the macrophage-specific genetic deletion of miR-21 inhibited cardiac fibrosis in a TAC model [15]. Ca^2+^-CaM–dependent signaling, such as NFAT dephosphorylation [16] and CaMKII signaling [17], is known to activate monocytes or cardiomyocytes and might be responsible for the secretion of growth factors or miR-21. Inhibition of Ca^2+^-CaM–dependent signaling in macrophages or cardiomyocytes may be a possible mechanism of DAN-mediated fibrosis inhibition. Elevated lung weight and a higher E/e′ suggest elevated LA pressure in the UNX + DOCA-salt group, and increased LV chamber stiffness might be responsible for the elevated LA pressure. Oral administration of DAN decreased LV chamber stiffness, resulting in decreased E/e′ and lung weight.

### 4.3 DAN inhibited nuclear translocation of CaM and thus suppressed cardiomyocyte hypertrophy

We previously showed that the angiotensin II–induced stimulation of isolated cardiomyocytes resulted in a decrease in RyR2-bound CaM and an increase in nuclear CaM, along with a decrease in the nuclear/cytoplasmic ratio of HDAC5 [5]. HDAC5 is known to negatively regulate the transcription factor myocyte enhancer factor 2 (MEF2) [18]; therefore, HDAC5 nuclear export activates MEF2-dependent gene transcription. Interestingly, the nuclear translocation of CaM has been shown to be inhibited by DAN [5]. In this regard, we have previously hypothesized that the dissociation of CaM from RyR2 plays an important role in the activation of hypertrophic signals. To prove this hypothesis, we used a knock-in (KI) mice model [19] in which single point mutation (V3599K) was applied at CaM binding domain in RyR2 to increase the binding affinity of CaM to RyR2. In this KI mice model, cardiac hypertrophy was not induced by transverse aortic occlusion [6]. Consistent with these data, pharmacological prevention of CaM dissociation from RyR2 by DAN prevented the development of LV hypertrophy in the present study.

## 5Conclusion

In conclusion, the oral administration of DAN to mineralocorticoid-salt–induced hypertensive rats inhibited cardiomyocyte hypertrophy and fibrosis and improved both LV relaxation and chamber stiffness. As a result, lung congestion was also improved. Therefore, DAN may be a novel therapeutic agent for HFpEF.

## Funding

This work was supported by Grants-in-Aid for 10.13039/100009950Scientific Research from the Ministry of Education, Culture, Sports, Science and Technology in Japan (grant Nos. 20H03677 to MY and 21K08054 to TY).

## Data availability

The experimental data will be shared on reasonable request to the corresponding author.

## Declaration of competing interest

The authors declare that they have no known competing financial interests or personal relationships that could have appeared to influence the work reported in this paper.
